# Temporal dynamics of HIV-1 circulating subtypes in distinct exposure categories in southern Brazil

**DOI:** 10.1186/1743-422X-9-306

**Published:** 2012-12-12

**Authors:** Sabrina EM Almeida, Rubia M de Medeiros, Dennis M Junqueira, Tiago Gräf, Caroline PB Passaes, Gonzalo Bello, Mariza G Morgado, Monick L Guimarães

**Affiliations:** 1 Centro de Desenvolvimento Técnico e Científico – CDCT, Fundação Estadual de Produção e Pesquisa em Saúde – FEPPS, Av. Ipiranga, 5400, 3° andar, CEP: 90610-000, Porto Alegre, RS, Brazil; 2 Laboratório de AIDS & Imunologia Molecular. Instituto Oswaldo Cruz – FIOCRUZ. Av. Brasil 4365, 439 - Pavilhão Leonidas Deane, sala 413, CEP: 21040-900, Rio de Janeiro, RJ, Brazil; 3 Programa de Pós-graduação em Genética e Biologia Molecular, Departamento de Genética, Centro de Ciências Biológicas, Universidade Federal do Rio Grande do Sul (UFRGS), 9500 - Prédio 43323M, CEP:91501-970, Porto Alegre, RS, Brazil; 4 Programa de Pós-graduação em Biotecnologia e Biociências, Departamento de Microbiologia, Imunologia e Parasitologia, Centro de Ciências Biológicas, Universidade Federal de Santa Catarina (UFSC), Campus Universitário, CEP: 88040-970, Florianópolis, SC, Brazil

**Keywords:** HIV-1, Brazil, Subtypes, Exposure categories, Temporal dynamics

## Abstract

**Background:**

The HIV-1 epidemic in Brazil is predominantly driven by subtype B. However, in Brazilian Southern region subtype C prevails and a relatively high AIDS incidence rate is observed. The aim of the present study was to assess the temporal dynamics of HIV-1 subtypes circulating in patients from distinct exposure categories in Southern Brazil. For this purpose 166 HIV-1 samples collected at the years of 1998 (group I) and 2005–2008 (group II) were analyzed.

**Results:**

Analysis of group I revealed statistically significant (p < 0.05) associations between MSM and subtype B as well as between IDU and subtype C; while no statistical significant association between HIV-1 subtypes and exposure category was verified for group II. An overall temporal increase in the prevalence of subtype C and BC recombinants was observed in both HET and MSM populations, accompanied by a proportional decrease in the prevalence of the pure subtype B.

**Conclusions:**

The present study shows an association between HIV subtypes and exposure categories at the middle 1990s in Southern Brazil. Our findings suggest that MSM and IDU populations might have played a major role in the introduction and initial dissemination of subtypes B and C, respectively, in Southern Brazil. This study also suggests a trend towards homogenization of HIV-1 strains across distinct exposure categories as a consequence of an overall increase in the prevalence of subtype C and BC recombinants in both HET and MSM populations.

## Introduction

The main hallmark of the HIV-1 is an extraordinary evolution rate, which results in high molecular diversity and dynamism of the AIDS epidemic [1]. HIV-1 is classified in four groups and the group M, currently estimated to infect around 33 million people around the world, is subdivided in 9 subtypes (A–D, F–H, J, and K) and 54 circulating recombinant forms (CRFs) [1-3].

Since 1980, Brazil has registered 608,230 cases of AIDS, representing an overall prevalence of 0.6% in adult population [4]. The HIV-1 subtype B is the predominant variant in most of the Brazilian regions followed by subtype F1, subtype C and a large variety of BF1 and BC recombinant forms [4-10]. The distribution of HIV-1 subtypes, however, is not homogeneous across the country and a distinct HIV-1 molecular epidemiologic scenario is observed in the Southern region. Composed by the states of Rio Grande do Sul, Santa Catarina and Paraná, the Brazilian Southern region shows a remarkably high prevalence of subtype C and BC recombinant forms [11-21].

The HIV-1 molecular epidemiology in Porto Alegre, capital of the Rio Grande do Sul state, is characterized by a high prevalence of subtype C (~30-40%), subtype B (~30-45%) and the circulating recombinant form (CRF) 31_BC (~10-25%), and lower prevalence of unique recombinant forms (URFs) and subtype F1 [12,16-18,21]. The southern region of Brazil is not only characterized by a distinct subtype profile, but also by a relative high AIDS incidence rate. This is particularly evident for the city of Porto Alegre, showing the highest AIDS incidence rate among all the Brazilian capitals since 1997 [4]. The reported AIDS incidence in Porto Alegre in 2010 (99.8 cases per 100,000 habitants) was more than five times higher than the mean incidence for the whole country (17.9 cases per 100,000 habitants) [4]. In this city, almost 80% of the new AIDS cases registered in 2009 correspond to heterosexual (HET) individuals, approximately 10% to men who have sex with men (MSM) and another 10% to injection drug users (IDU) [22].

Some studies indicate a tendency towards an increasing proportion of subtype C infections over time in some cities from the Southern region. The estimated prevalence of subtype C increased from 36% before 1997 to 53% in 2008 in Rio Grande (Rio Grande do Sul state) [11,23], and from 56% to 78% between 2004 and 2009 in Florianopolis (Santa Catarina state) [14]. Further studies also point to a different spreading of the HIV-1 variants among the exposure categories in southern Brazil. Studies conducted in all the three states of Brazilian Southern region described that subtype C is more frequently seen in the HET population, while subtype B is more common in MSM [14,20,23]. One study also found an association between subtype C infections and the use of intravenous drugs among males in the state of Parana [15]. So far, no significant associations between HIV-1 clades and exposure categories were detected in the city of Porto Alegre, the main capital of Southern Brazil, even though an association of subtype B with anal sex practices and a tendency of subtype C with females have been reported [11,17,21].

Most of the previous studies that analyzed the temporal dynamics of HIV-1 diversity in Southern Brazil were based on the stratification of cross-sectional samples according to time of HIV diagnosis and without taking into account the exposure categories of the individuals. In this study, we described the temporal trends of HIV-1 clades in patients of different exposure categories living in the city of Porto Alegre, through the analysis of two groups of samples collected at 1998 and 2005–2008.

## Methods

### Study population

Blood samples from 166 HIV-positive patients followed up at different outpatients clinics in the metropolitan region of Porto Alegre, the capital of the Southernmost state of Brazil, were collected at two different time periods: 1998 (n = 83) and 2005–2008 (n = 83). The inclusion criteria for individuals in both studied periods were age over 18-year old, agreement to participate in the study, read and signed an informed consent form. In the second period the individuals had to report no previous antiretroviral therapy. The clinical and demographic data of the patients (age, sex, first positive serology for HIV-1 and CD4+ T-cell counts) are shown in Table 1. This study was approved by the Ethical Research Committee from Fundação Estadual de Produção e Pesquisa em Saúde number18/2005.

**Table 1:** Clinical and demographic data of the HIV-positive patients from Porto Alegre at two distinct time periods - 1998 (group I) and 2005–2008 (group II)

	**Group I (n=83)**	**Group II (n=83)**
**Age (years)**	33 ± 10	35 ± 10
**Gender**		
**Male**	59 (71.0%)	49 (59.3%)
**Female**	24 (29.0%)	34 (40.7%)
**CD4 T cell count**		
(cell/mm3)*		
**200**	23 (29,1%)	16 (28,6%)
**200-400**	25 (31,6%)	13 (24,1%)
**>400**	31 (39,2%)	27 (50,0%)
**Exposure category**		
**HET**	42 (50.6%)	61 (73.5%)
**MSM**	32 (38.6%)	22 (26.5%)
**IDU**	9 (10.8%)	-
**First positive serology for HIV (years)**	1994 [1989–1998]	2005 [2002–2008]

* information not available for four individuals from group I and twenty seven from group II. HET – heterosexuals; MSM – men who have sex with men; IDU – injection drug users.

### HIV-1 amplification, sequencing and subtyping

DNA samples were extracted from 200 μl of whole blood using a QIAamp DNA kit (Qiagen Inc., CA, U.S.A.), according to the manufacturer’s protocol. Amplification and sequencing of the PR/RT region was performed as described elsewhere [24]. The sequences generated were ~1,160 nt long and covered the protease (PR) and part of the reverse transcriptase (RT) genes (nucleotides 2253–3413 relative to HXB2).

Nucleotide sequences were aligned using the Clustal X program [25] and three strategies were used to well characterize the HIV-1 sequences as pure subtype, CRF-like or URF using the pol alignment: i) a Neighbor-Joining (NJ) phylogenetic tree was first built under the Tamura-Nei substitution model in 1000 bootstrapped data sets, as implemented in MEGA program [26]; ii) all sequences were subsequently subjected to bootscanning with the Simplot software version 3.5.1 [27], using reference sequences representative of HIV-1 subtypes A1, B, C, and F1 available in Los Alamos database, http://www.hiv.lanl.gov/components/sequence/HIV/search/search.html). Bootstrap values supporting branching with reference sequences were determined in NJ trees constructed using the K2-parameter model [28], based on 100 re-samplings, with a 300 nt sliding window moving in steps of 10 bases; iii) to better characterize the recombination breakpoints suggested in the previous analyses, the putative recombinants were subjected to informative site analyses as described elsewhere [24]. Consensus sequences used in our analyses were generated from a total of 491 subtype B and 164 subtype C pol Brazilian sequences downloaded from the Los Alamos HIV Sequence Database, as implemented in the DAMBE program [29]. Sequences were submitted to GeneBank under the following accession numbers: [JF487830 - JF487913] and [JQ619540 - JQ619621].

### Statistical analyzes

Statistical comparisons among subtypes groups and exposure categories were made using Pearson’s χ^2^-test with adjusted residues and Fisher’s exact test when appropriate. Statistical analysis was performed using the SPSS 16.0 statistical package and the significance level was set at p<0.05.

## Results

Group I was composed by HIV-1 positive samples collected in 1998 from 83 individuals, of which 71.0% were men. Based on medical records, patients were classified into three exposure categories: HET (50.6%), MSM (38.6%) and IDU (10.8%). The mean diagnostic year of patients from group I was 1994 (Table 1). Group II, comprised 83 HIV-1 positive individuals sampled from 2005 to 2008, of which 59.3% were men (Table 1). Regarding the exposure categories, group II showed 73.5% of HET individuals and 26.5% of MSM, the mean diagnostic year being 2005. Most patients of both groups (39,2% and 50%, respectively) presented T CD4 cells counts above 400cell/mm3. All patients included in group II were antiretroviral naïve, while 55% of individuals from group I declared to be under antiretroviral therapy.

According to the phylogenetic, bootscanning, and informative site analyses of the PR/RT region, the 166 HIV-1 samples from Porto Alegre here analyzed were classified as follows: subtype B (n = 65), subtype C (n = 37), CRF31_BC (n = 28), URFs_BC (n = 25), subtype F1 (n = 3), URFs_BF (n = 5), and URF_BCF (n = 3) (Additional file 1: Figure S1). Some of the URFs_BC detected in the present study (n = 13), shared one of the recombination breakpoints with the CRF31_BC and were probably created by the recombination of the CRF31_BC with local subtypes C and B. Since CRF31_BC and the URFs_BC with a CRF31_BC-related structure probably share a common evolutionary history, they were grouped together for further analyses. Other URFs_BC with a mosaic structure not related to the CRF31_BC (n = 12) were allocated in a separate group. The recombination pattern of all URFs and a schematic draw of the CRF31_BC-related structure are provided as Additional file (Additional file 2: Figure S2).

The analyses of HIV diversity and exposure categories for both time periods are described in Table 2. The analysis of HIV-1 clades for group I revealed a significant association (p < 0.05) between MSM exposure category and subtype B, as well as between IDU patients and subtype C. The HIV-1 subtype B variant was responsible for more than 70% of infections in MSM individuals, while it represented 50% of infections in HET and only 11% of infections among IDU. By contrast, the HIV-1 subtype C variant was more frequent among IDU patients (44.4%) than among HET (14.3%) and MSM (6.2%) individuals. The frequency of URFs_BC in the IDU population (22.2%) was also higher than in the MSM (3%) and HET (0%) populations (p<0.05). The CRF31_BC and other BC recombinants with a related mosaic structure were more frequent in IDU (22.2%) and HET (21.4%) populations than in the MSM (9.4%) group, while subtype F1 and BF1 recombinants displayed a higher frequency in the HET (14.3%) and MSM (9.4%) populations compared to IDU (0%), although those differences were not statistically significant.

**Table 2:** HIV-1 subtype frequencies according to the patient’s exposure category

		**Exposure Category**		
		**HET**	**MSM**	**IDU**
**Group I**				
	Subtype B	21 (50.0)	23 (72.0)*	1 (11.0)
	Subtype C	6 (14.3)	2 (6.2)	4 (44.6)*
	CRF31_BC and related recombinants	9 (21.4)	3 (9.4)	2 (22.2)
	URFs_BC	-	1 (3.0)	2 (22.2)*
	Subtype F1 and URF_BF	6 (14.3)	3 (9.4)	-
	**Total (n=83)**	42	32	9
**Group II**				
	Subtype B	13 (21.4)	7 (31.8)	-
	Subtype C	21 (34.4)	4 (18.2)	-
	CRF31_BC and related recombinants	22 (36.0)	5 (22.7)	-
	URFs_BC	4 (6.6)	5 (22.7)	-
	Subtype F1 and URF_BF/URF_BCF	1 (1.6)	1 (4.5)	-
	**Total (n=83)**	61	22	-

*significant p value <0.05. Values between brackets are relative percentages of the total of the column. HET – heterosexuals; MSM – men who have sex with men; IDU – injection drug users.

Analysis of the group II reveals no significant association between HIV-1 clades and the exposure category, even though some differences in the most prevalent clades circulating in HET and MSM individuals were evident. Most HIV-1 infections in the HET population were associated to subtype C (34.4%) and CRF31_BC and related recombinants (36%), followed by subtype B (21.4%) and URFs_BC (6.6%). By contrast, the majority of HIV-1 infections detected in the MSM population were related to subtype B (31.8%), followed by roughly similar frequencies of CRF31_BC and related recombinants (22.7%), URFs_BC (22.7%), and subtype C (18.2%). In both groups, the frequency of subtype F1 and URFs_BF/URFs_BCF was very low (<5%).

**Figure 1:**
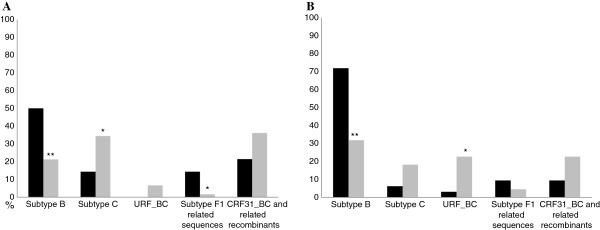
**Prevalence of HIV-1 subtypes in Porto Alegre in the two studied populations (1998 and 2005–2008) according to exposure categories (A) Heterosexual and (B) Men who have sex with men.** Black bars represent Group I and gray bars represent Group II. Symbols represent statistical significance, * = p<0.05; ** = p<0.01.

The analysis of the temporal dynamics of HIV-1 clades circulating in the HET and MSM individuals revealed a significant (p<0.01) decreasing in prevalence of subtype B in both exposure categories over time (Figure 1). In 1998, 50% of HET and 72% of MSM individuals were infected by subtype B, passing to 21% and 32% in 2005–2008, respectively. At the same time, the prevalence of subtype C, CRF31_BC and related recombinants and URFs_BC increased in both HET and MSM individuals, although only the increase of subtype C in HET (14% to 34%) and of URFs_BC in MSM (3% to 23%) were statistically significant (p<0.05) (Figure 1). A decrease in the prevalence of subtype F1 and URFs_BF was also observed and it was more pronounced in the HET population, in which the frequency of those clades decreased from 14% to 2% (p<0.05) (Figure 1). When the HET group was analyzed by gender, it was observed a significant (p<0.05) increase of subtype C (8.3% to 33.3%) across time in women, significant (p<0.05) decrease of subtype B in both women (50% to 24.2%) and men (50% to 17.9%) and significant (p<0.05) decrease of subtype F1 and URFs_BF (12.5% to 0%) in women.

## Discussion

The current study compared the HIV-1 molecular epidemiologic scenario at two distinct time periods in the city of Porto Alegre, Southern Brazil. Patients from group I, recruited in 1998 were diagnosed between 1986 to 1998 and were likely infected around the early 1990s. Patients from group II, recruited between 2005 and 2008 were diagnosed between 2002 to 2008, and constitute a picture of the HIV-1 epidemic around the early to middle 2000s. Given the broad scope of these two sample groups, the analyses performed here have a temporal feature, shedding some light to the origins of the singular HIV-1 epidemic in Southern Brazil.

**Figure 2:**
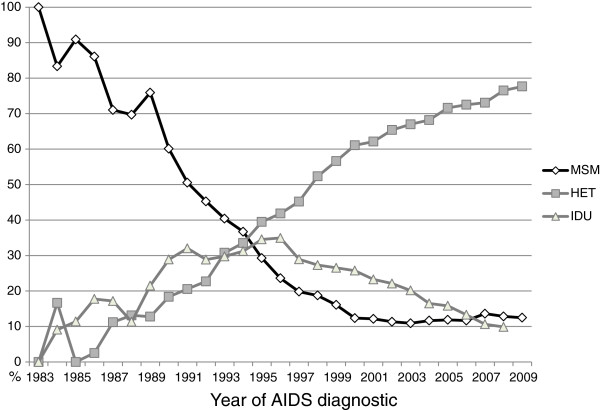
**Proportion of AIDS diagnosed individuals according to exposure categories in Porto Alegre – Brazil.** Black line with diamonds represents Men who have sex with men, grey line with squares represents heterosexuals and gray line with triangles represents injection drug users. Source: http://www.aids.gov.br/pagina/tabulacao-de-dados - access October 2011.

Analysis of the group I revealed a significant association between the MSM exposure category and subtype B, as well as between IDU patients and subtype C. The MSM and IDU population have played a major role in the dynamics of the HIV epidemic in Porto Alegre, particularly up to the middle 90s (Figure 2). Although harm reduction policies promoted by the Brazilian Ministry of Health reduced the proportion of AIDS cases in IDU from more than 25% before 1990 to around 5% in 2010, Porto Alegre continues to figure as the Brazilian city with the highest number of AIDS cases among IDU [4,22]. No temporal analysis of the epidemic in the IDU exposure category was performed as a consequence of the absence of IDU in group II. Selecting a sample of IDU is not a simple task since injecting drug is an illegal and stigmatizing behavior, and these individuals are usually outside the public health services. These results suggest that HIV-1 clades B and C may have been introduced and initially disseminated in Porto Alegre through the MSM and IDU transmission networks, respectively. Similar findings have been recently reported in other Southern Brazilian states. One study reported an association between subtype C infections and the use of intravenous drugs among males in the state of Parana [15], while other studies described an association between subtype B infections and the MSM population in Parana, Santa Catarina and Rio Grande do Sul states [14,20,23].

The proposed scenario is consistent with the proposed evolutionary history of subtype B and C epidemics in Brazil. Some studies suggest that the pandemic subtype B clade was introduced in Brazil around 1965–1970, at least 10–15 years earlier than subtype C [30,31]. Thus, the pandemic subtype B is the most probable HIV-1 clade responsible for the initial AIDS cases described in Brazil, including the city of Porto Alegre, during the early 1980s, that mostly affect the MSM population (Figure 2). The proportion of AIDS cases corresponding to IDU and HET individuals started to growth in Porto Alegre around the middle 1980s (Figure 2), shortly after the estimated introduction of subtype C clade [32]. Of note, the estimated onset date of the CRF31_BC clade (around the late 1980s) [24], coincides with a peak in the proportion of AIDS cases due to IDU infections in Porto Alegre (Figure 2). Since HIV transmission is very efficient through injecting equipment, super-infections with distinct viral forms are constant among IDU [33], which may have allowed the emergence of diverse recombinants forms, including the CRF31_BC.

Analysis of the group II reveals that by the middle 2000s most HIV-1 infections in the HET population were associated to subtype C (34%) and CRF31_BC and related recombinants (36%), while subtype B was the most common genetic variant in the MSM population (32%). However, no significant association between HIV-1 clades and the exposure infection risk were detected at this second time point. This coincides with other studies conducted in Porto Alegre between 2002 and 2009, that also have failed to find a significant association between HIV-1 clades and the exposure category [17,21]. This lack of association may be explained by a progressive intermixing and homogenization of HIV-1 clades between different transmission networks in Porto Alegre over time, characteristic of a mature epidemic.

Comparison between groups I and II revealed a temporal increase in the proportion of subtype C (from 14% to 34%) and CRF31_BC and related recombinants (from 21% to 36%) in HET individuals. This is consistent with a previous coalescent analysis that supports an exponential expansion of subtype C and CRF31_BC clades in Porto Alegre during the 1980s and 1990s [34]. This expansion also coincides with the growing proportion of AIDS cases in IDU and, particularly, in the HET population in Porto Alegre (Figure 2), in agreement with the process of feminization of the AIDS epidemic observed throughout the country [4]. Similar to the AIDS Brazilian epidemic, the present study also observed an increase in heterosexual transmissions (50.6%-73.5%) and in female cases (29.0%- 40.7%) between the two groups of patients (Table 1). It is conceivable that shortly after introduction into IDU population, subtype C may have passed to HET. The early introduction of subtype C into the HET group may have promoted the successful spread of this variant in Porto Alegre, even after the decrease of HIV infections among IDU.

An increase in the proportion of subtype C (from 6% to 18%) and CRF31_BC and related recombinants (from 9% to 23%) between the two time periods was also evident among MSM individuals from Porto Alegre, thus providing evidence that dissemination of subtype C and CRF31_BC was not limited to the HET group. These results support the hypothesis that some associations between HIV-1 strains and sexual exposure categories detected in Brazil may be due to founder effects, rather than to a different efficacy of transmission of subtypes B and C through MSM or HET individuals. Analysis of the MSM population further reveals a significant increase in the frequency of URFs_BC (from 3% to 23%), that could be explained by the gradual dissemination of subtype C into this group coupled to high rates of coinfection and/or superinfection. Bisexual men and homosexual IDU might have played an essential role in this process, promoting a linkage between partially isolated transmission networks.

The overall increase in the prevalence of subtype C and BC recombinants in Porto Alegre was accompanied by a proportional decrease in the prevalence of subtype B in both HET (from 50% to 21%) and MSM (from 72% to 32%) populations. One hypothesis to explain this result is that subtype C displays a higher sexual transmissibility than subtype B. Alternatively, the dissimilar outcome of subtypes B and C in the city of Porto Alegre may be a consequence of differences in the transmission networks that promoted the initial dissemination of each subtype in the city. It is possible that subtype C and the CRF31_BC gained access to large networks of IDU and HET groups before subtype B, and this may have conditioned the subsequent dissemination of those clades among all different exposure categories. The great variation in the relative prevalence of clades B and C across different cities in Southern Brazil favors this second hypothesis. Of note, interpretations of the observed patterns should be considered with caution because of the limited sample size and the absence of IDU in the second casuistic. Future studies including larger number of HIV+ patients within different exposure categories will be necessary to confirm the observed trends.

## Conclusions

The data presented here points to a possible introduction of subtype B and C through different transmission networks in the city of Porto Alegre. Subtype B was probably introduced through the MSM individuals while subtype C may have been introduced into the IDU network and rapidly disseminated to the HET group. This study also suggests a trend towards homogenization of HIV-1 strains across distinct exposure categories as there has been an overall increase in the prevalence of subtype C and BC recombinants in both HET and MSM populations. Understanding the mechanism responsible for the expansion and contraction of subtypes C and B, respectively, in Porto Alegre, is of paramount importance to understand the HIV-1 dynamics in this country region.

## Abbreviations

AIDS: Acquired immune deficiency syndrome; CRF: Circulating recombinant form; IDU: Injection drug users; HIV: Human immunodeficiency virus; HET: Heterosexual; MSM: Men who have sex with men; NJ: Neighbor-joining; PR: Protease; RT: Reverse transcriptase; URF: Unique recombinant form.

## Competing interests

The authors declare that they have no competing interests.

## Authors’ contribution

SEMA designed the study, helped to draft the manuscript and coordinated the statistical analysis. RMM, DMJ and CPBP collected the samples, carried out the molecular studies and the phylogenetic analysis. TG drafted the manuscript and performed the statistical analysis. GB participated in the study design and helped in the phylogenetic and statistical analysis. MGM helped in the draft of the manuscript. MLG conceived of the study, participated in its design and coordination and edited the manuscript. All authors read and approved the final manuscript.

## Supplementary Material

Additional file 1 **Figure S1.** Neighbor-Joining phylogenetic tree with Tamura-Nei substitution model of HIV-1 PR/RT region (2253–3413 relative to HXB2) of samples from group I and II. Only “pure” subtype C, B, F1 and CRF31_BC were included. Bootstrap values above 80% obtained for 1000 replicates are shown in the nodes. Click here for file

Additional file 2 **Figure S2.** Schematic drawing showing breakpoint pattern of the URF viruses found in the study. Breakpoint positions were obtained using Simplot 3.5.1 and numbered according to HXB2 reference. Sequences CRF31_BC related are characterized by the presence of a slightly smaller or bigger subtype B fragment. Click here for file
