# Supplementary figures and images for: Temporal dynamics of HIV-1 circulating subtypes in distinct exposure categories in southern Brazil
Source: Virol J. 2012 Dec 12;9:306. doi: 10.1186/1743-422X-9-306 (PMC3547702; doi:10.1186/1743-422X-9-306)

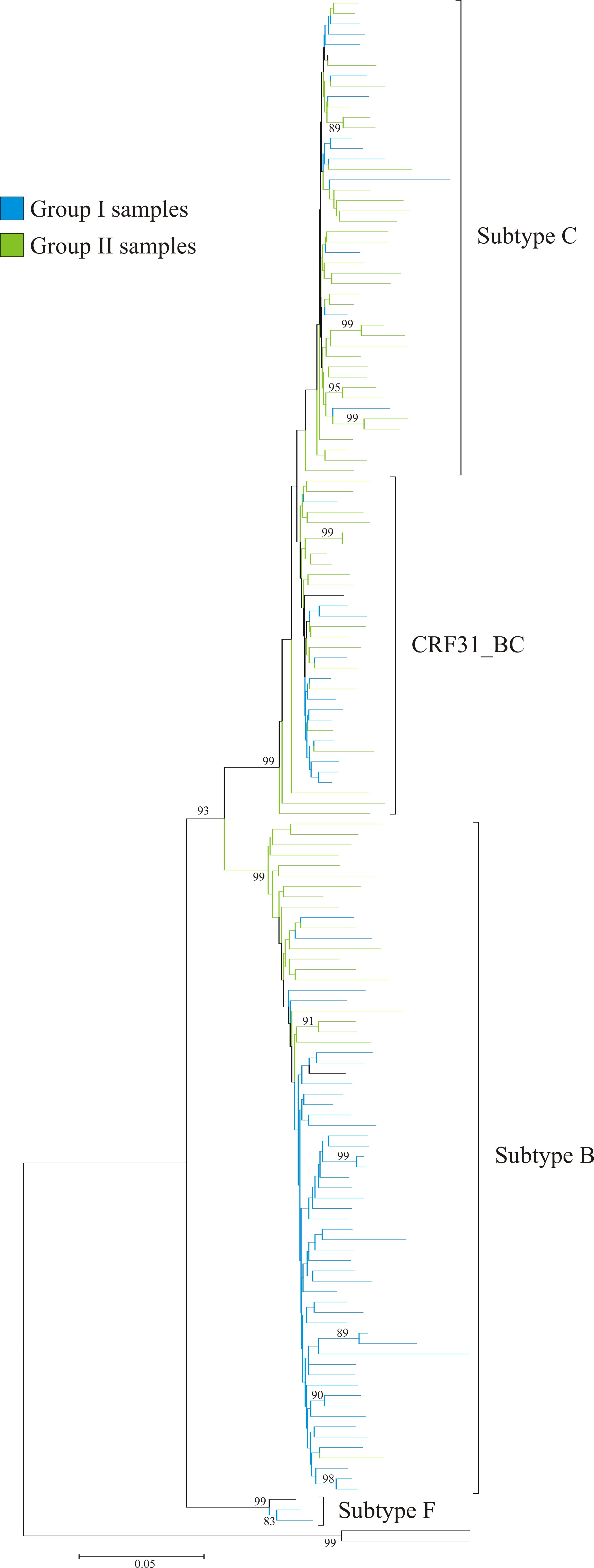

Supplement: Additional file 1 — Figure S1. Neighbor-Joining phylogenetic tree with Tamura-Nei substitution model of HIV-1 PR/RT region (2253–3413 relative to HXB2) of samples from group I and II. Only “pure” subtype C, B, F1 and CRF31_BC were included. Bootstrap values above 80% obtained for 1000 replicates are shown in the nodes. [file 1743-422X-9-306-S1.jpeg]

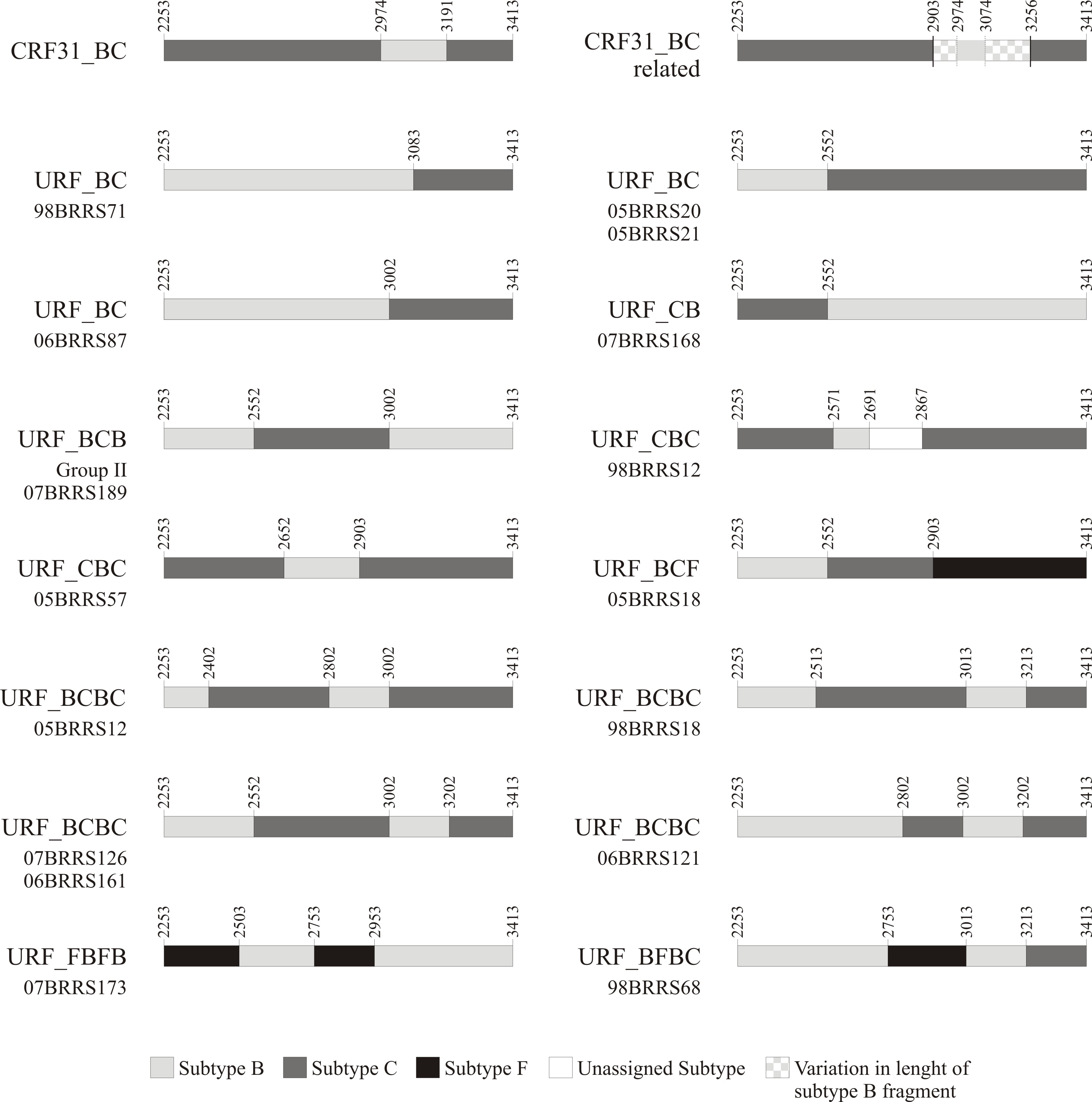

Supplement: Additional file 2 — Figure S2. Schematic drawing showing breakpoint pattern of the URF viruses found in the study. Breakpoint positions were obtained using Simplot 3.5.1 and numbered according to HXB2 reference. Sequences CRF31_BC related are characterized by the presence of a slightly smaller or bigger subtype B fragment. [file 1743-422X-9-306-S2.jpeg]
